# Development of a Blended Physical Activity Intervention for Office Employees Using Intervention Mapping: Intervention Development Study

**DOI:** 10.2196/87328

**Published:** 2026-07-14

**Authors:** Yan Sun, Alison YT Ou, Tony Lei Shi, Yang Gao, Martin S Hagger, Zhennan Xiong, Xiang-yan Chen, Shirley SM Fong, Linda L.D. Zhong, Yao Jie Xie, Julien S. Baker

**Affiliations:** 1 Academy of Wellness and Human Development Faculty of Arts and Social Sciences Hong Kong Baptist University Hong Kong China (Hong Kong); 2 Chinese University of Hong Kong Hong Kong China (Hong Kong); 3 Faculty of Arts and Social Sciences Hong Kong Baptist University Hong Kong China (Hong Kong); 4 School of General Education Beijing Normal-Hong Kong Baptist University Zhu Hai, Guangdong China; 5 Department of Psychological Science University of California, Merced Merced, CA United States; 6 Health Sciences Research Institute University of California, Merced Merced, CA United States; 7 School of Applied Psychology Griffith University Brisbane, Mount Gravatt Australia; 8 Faculty of Sport and Health Sciences University of Jyväskylä Jyväskylä Finland; 9 Division of Science, Engineering and Health Studies (SEHS) The Hong Kong Polytechnic University College of Professional and Continuing Education Hong Kong China (Hong Kong); 10 Department of Health and Physical Education The Education University of Hong Kong Hong Kong China (Hong Kong); 11 School of Biological Sciences Nanyang Technological University Singapore Singapore; 12 School of Nursing The Hong Kong Polytechnic University Hong Kong China (Hong Kong); 13 Neurology and Endocrinology Departments Medical Research Centre Ningbo No.2 Hospital Ningbo, Zhejiang China

**Keywords:** physical activity, intervention mapping, behavior change, theory and evidence-based, intervention development, intervention design

## Abstract

**Background:**

Insufficient engagement in moderate to vigorous physical activity (MVPA) is a significant risk factor for major noncommunicable diseases, including cardiovascular diseases (eg, coronary heart disease and stroke), type 2 diabetes, and several cancers. Physical inactivity accounts for an estimated 3.2 million deaths annually. Office employees, due to their sedentary and desk-based work patterns, are particularly vulnerable to low MVPA levels, which negatively affect health and work productivity. The COVID-19 pandemic exacerbated these issues, further reducing MVPA levels due to lockdowns and work-from-home policies. Although numerous interventions have aimed to promote MVPA, many lack theoretical grounding, stakeholder involvement, or systematic development frameworks. A theory- and evidence-informed approach is warranted to target modifiable determinants and mechanisms, improve coherence and replicability, and enhance effectiveness and scalability.

**Objective:**

This study aimed to design an evidence-based blended intervention to improve MVPA, health, and productivity among office employees, guided by the intervention mapping (IM) framework.

**Methods:**

Following the first 4 steps of the IM framework, we developed a 12-week blended intervention and created a website named “Smartly Active for Health and Life.” Step 1 involved a needs assessment through a literature review and focus group discussions (n=12 office employees) to identify barriers, facilitators, and determinants of MVPA. Step 2 defined intervention outcomes, performance objectives, and change objectives based on the social ecological model. Step 3 integrated theory- and evidence-based behavior change techniques with delivery methods, resulting in a 3-group randomized controlled trial design: (1) a blended intervention group (web-based sessions and e-workshops), (2) a web-only intervention group, and (3) an active control group. Step 4 developed and refined intervention materials and protocols through a pilot study (n=18). Key behavior change techniques included problem-solving, goal setting, self-monitoring, and habit formation.

**Results:**

The pilot study demonstrated the intervention’s acceptability, feasibility, and adaptability. Participants reported increased motivation, improved knowledge of MVPA benefits, and greater confidence in goal setting and self-regulation. Feedback led to refinements such as improved website navigation and interactive content. The intervention is expected to enhance MVPA levels, health, and work productivity by addressing both motivational and volitional phases of behavior change while supporting habitual behaviors.

**Conclusions:**

The IM framework offered a systematic and iterative approach for developing a blended MVPA intervention tailored to the needs of office employees. This study highlights the importance of theory-driven and stakeholder-informed approaches in designing scalable and sustainable health promotion programs. The intervention has the potential to address the critical public health challenge of physical inactivity in office settings and serves as a model for future behavior change interventions.

**International Registered Report Identifier (IRRID):**

RR2-10.1186/s12889-020-09128-z

## Introduction

Physical inactivity is a leading risk factor for global mortality, contributing significantly to common noncommunicable diseases and increased health care burdens [1]. Regular moderate to vigorous physical activity (MVPA) mitigates such risks, improving physical and mental health, work productivity, and overall well-being [2-4]. World Health Organization (WHO) guidelines recommend that adults aged 18 to 64 years engage in 150 to 300 minutes of MVPA per week, but approximately 1.8 billion adults (31% of the global adult population) do not achieve this recommendation [5,6]. A systematic review revealed that working adults engage in an average of 14.7 minutes of MVPA daily, with office employees exhibiting the most sedentary behavior due to their desk-based work and daily tasks [7]. In the COVID-19 pandemic (2020 to 2022), there was a marked decrease in adults’ MVPA levels due to lockdowns, closures, and limited mobility, with office employees particularly affected by work-from-home setups, leading to reductions in commuting and leisure activities [8-10].

Many theory-based and non–theory-based interventions have been developed to increase MVPA levels among adults. However, the effectiveness of such interventions remains unclear due to the substantial heterogeneity in their designs and components [11]. Furthermore, many interventions have been developed without reference to sufficient empirical evidence and have frequently failed to include key stakeholders’ perspectives [12]. There is evidence that behavioral change interventions based on theoretical frameworks, empirical evidence, and stakeholder engagement are potentially more effective than those lacking a theoretical basis [13-15]. Nevertheless, few well-designed theory-based interventions have been conclusively demonstrated to be effective.

Intervention mapping (IM) is regarded as a systematic framework that guides effective decision-making throughout the development, implementation, and evaluation of evidence-based health promotion and disease prevention programs. It is a stepwise process that uses theory, empirical evidence, and community feedback to develop interventions that are effective, feasible, sustainable, and customized to the needs of the target population [16]. The six steps of IM comprise (1) conducting a needs assessment; (2) specification of intervention outcomes, objectives, and change objectives; (3) selection of theory- and evidence-based change methods and practical modes of delivery; (4) creation and refining of materials and a protocol; (5) development of an implementation plan; and (6) design of an evaluation plan.

There is an urgent need for interventions to increase MVPA levels using the IM framework to bridge existing research gaps and enrich the evidence base. While the protocol and results of the main randomized controlled trial (RCT) examining the effectiveness of the intervention have been published elsewhere [17,18], this paper focuses on the development process of the blended intervention. Specifically, it describes the application of IM steps 1 to 4 (needs assessment, intervention outcomes and objectives, intervention design, and intervention production). The final stages of the IM framework, intervention implementation and evaluation, will be detailed in separate publications.

## Methods

### Overview

Steps 1 to 4 in this paper involved developing and refining the intervention protocol and materials, with each step building on the results of the previous step. A book on the IM approach [16], which outlines the planning of health promotion programs, served as the foundation for the development of the intervention. The methodology and tasks of steps 1 to 4 are detailed in Multimedia Appendix 1.

### Step 1: Needs Assessment

The first step of the IM framework involved a needs assessment to identify the health problem, justify the promotion of MVPA, and determine the underlying determinants in the target population. This provided a solid foundation for designing an evidence-based and culturally appropriate intervention [16].

#### Planning Group

A planning group was formed at the start of the process, comprising researchers with expertise in epidemiology, statistics, health promotion, psychology, and sports science, as well as office employees from local companies. These stakeholders ensured all steps of the intervention were well aligned with workplace realities. Weekly meetings focused on planning and brainstorming, transitioning to biweekly sessions for progress tracking. However, due to challenges presented by the COVID-19 pandemic restrictions, managers had to be excluded from the group.

#### Literature Review

A comprehensive literature review was conducted to better understand the prevalence and effects of insufficient MVPA on health and workplace productivity, as well as the most common barriers and facilitators identified in working adults. The review also explored the evidence on theory- and evidence-based change methods and delivery modes for intervention design, which has been published elsewhere [19]. Six academic databases (Scopus, PsycInfo, PubMed, Embase, Web of Science, and SPORTDiscus) were searched for original studies and systematic reviews published in English before July 30, 2022. Search terms included topics like “physical activity,” “working populations,” “health outcomes,” and “theory-based interventions.” Governmental reports from organizations such as the WHO were also consulted to provide global context. Detailed search strategies can be found in Multimedia Appendix 2.

#### Focus Group Discussions

Focus group discussions were conducted to complement findings from the literature review and gain detailed insights into the barriers, facilitators, and preferences for interventions specific to insufficiently active office employees. This group was intentionally selected to ensure the study directly addressed the needs of individuals likely to benefit most from tailored interventions. Two focus groups (n=12) were conducted to explore gender-specific barriers and facilitators to MVPA [20,21]. Participants were intentionally recruited via online surveys. Of the 12 participants, 6 (50%) were female, with a mean age of 29 (SD 3.08) years. A total of 9 (75%) held a bachelor’s degree or higher, and 3 (25%) had a diploma or lower educational qualification. The participants provided informed consent to be video recorded during discussions. The focus groups, moderated via Zoom (Zoom Video Communications, Inc) due to COVID-19 pandemic restrictions, lasted 60 to 100 minutes and were facilitated by a trained qualitative researcher, with a second researcher present for note-taking and follow-up questions. The video recordings were transcribed verbatim by 2 trained investigators (AYTO and YS). The transcripts were cross-checked with the video recordings to ensure accuracy. The transcripts were reviewed and analyzed, and the research questions were used as a framework to categorize codes and derive themes from the narrative data [22]. Any discrepancies in interpretation were collaboratively discussed until a consensus was reached.

### Step 2: Intervention Outcomes and Objectives

Step 2 focused on defining the desired intervention outcomes and specifying performance objectives (POs) based on the needs assessment conducted in step 1. The planning group developed a matrix of behavioral change objectives by linking these POs with the underlying determinants of MVPA identified earlier, ensuring that the intervention addressed the key factors influencing MVPA behavior.

### Step 3: Intervention Design

The purpose of step 3 was to design theory- and evidence-based interventions to promote MVPA, addressing the determinants and objectives identified in step 2. This step involved selecting behavior change methods, linking them to practical applications, and specifying their implementation. The effective theories, behavior change techniques (BCTs; eg, goal setting and self-monitoring), and delivery modes (eg, web-based platforms) were identified from the literature review [19,23,24]. These findings informed the intervention’s design to ensure alignment with evidence-based practices. The planning group held additional meetings to finalize the intervention’s structure, components, and sequence, ensuring feasibility and cultural relevance within workplace settings.

### Step 4: Intervention Production

Step 4 focused on preparing the intervention materials, messages, and activities, followed by a 12-week pilot study with 18 participants (6 per group, excluded from the main study) to evaluate the readability of the materials, message execution, and acceptability, feasibility, and adaptability of the intervention. The pilot study also assessed participant recruitment and outcome measures, while collecting feedback from participants and implementers. On the basis of the pilot results, the intervention materials, protocols, and implementation plans were refined to optimize delivery and ensure smooth execution.

The original study protocol [17], developed before the COVID-19 pandemic, had to be revised due to challenges caused by the pandemic. Between June and August 2022, despite reaching out to approximately 2000 companies, none agreed to participate, mainly due to concerns about COVID-19 transmission and disruptions in work routines. As a result, the original cluster-RCT design was replaced with an individualized RCT. Key changes to the study design are summarized in Multimedia Appendix 3.

### Ethical Considerations

This study received ethics approval from the Research Ethics Committee of Hong Kong Baptist University (HASC/17-18/0529). Electronic informed consent was obtained from the 18 participants included in the pilot study after they were informed of the study objectives, procedures, potential risks and benefits, data use, confidentiality protections, and their right to withdraw. Identifiable information was stored separately from research data and will be destroyed after study completion. Deidentified datasets will be retained for research purposes and may be available from the corresponding author upon reasonable request. No identifiable participant information or images are included in this manuscript or supplementary materials. Participants received HK $50 (HK $1=US $0.13 as of June 17, 2026) coupons for each completed evaluation session and a personalized physical activity and health tracking report as part of the study benefits.

## Results

### Step 1: Needs Assessment

The literature review analyzed 183 articles spanning observational, intervention-based, and systematic review studies. Key themes identified barriers and facilitators to MVPA participation, categorized into personal, interpersonal, and environmental levels following the social ecological model [16]. These insights, outlined in Table 1, were enriched by focus group discussions to create a tailored intervention addressing the needs of inactive office employees.

**Table 1:** Facilitators of and barriers to moderate to vigorous physical activity (MVPA) participation identified from literature reviews and focus groups.

	Facilitators of MVPA participation	Barriers to MVPA participation
Personal level [25-30]	Knowledge of the benefits of MVPA (physical and mental health and disease prevention) Self-monitoring (eg, track progress and record workouts) Time availability (time management) Self-efficacy and confidence Improve sleep quality Improve self-esteem and sense of achievement Workplace well-being and quality of life Being active with children	Lack of knowledge and awareness (importance of participating in MVPA) Lack of time or competing demands on time Lack of motivation (eg, lack of interest and low self-efficacy) Lack self-monitoring and goal setting Health limitations or concerns (eg, health conditions, physical limitations, and injuries) Psychological barriers (eg, mental health concerns, perceived lack of energy, fatigue, and concerns about sweating) Safety concerns Minimize exposure to infection during the COVID-19 pandemic Unpredictable breaks and shifts (eg, certain life changes or events)
Interpersonal level [25-27,31]	Body image and societal beauty standards Religious and cultural norms Mentoring and coaching (professional supervision) Social ties Positive support (social, emotional, and psychological)	Lack of social support Negative social influences (eg, peer pressure and lack of positive role models)
Environmental level [25,26,28,32]	Accessibility of resources (eg, facilities, supportive environments, and professional supervision) Leadership and institutional support Supportive policies (eg, reminders for work breaks)	Limited access to facilities, environment, and space Lack of professional-development opportunities Lack of institutional support Lack of financial resources Inclement weather (eg, rain and extreme heat) COVID-19 pandemic policy restrictions (eg, closure of sports venues and exercise facilities to reduce infection risk)
Additional findings from focus groups	Young, energetic, and confident Improve work productivity Master motor and athletic skills Interesting and engaging Incentives Policies and flexible work arrangements Environmental modifications	No additional information was added from focus groups.

### Step 2: Intervention Outcomes and Objectives

In step 2, outcomes and POs were defined, leading to the creation of a matrix of change objectives. The primary outcome was expected to be an increase in MVPA levels. The secondary outcomes were expected to be improvements in health and work productivity. The POs developed in Table 2 were aligned with the determinants identified in step 1 to construct a matrix of change objectives (Multimedia Appendix 4). For example, PO5 (Table 3) focuses on action planning and highlights determinants such as knowledge, self-efficacy, and social support.

**Table 2:** Performance objectives (POs) for promoting moderate to vigorous physical activity (MVPA) levels among inactive office employees.

Code	Performance objective
PO1	Possess knowledge of MVPA and its impacts
PO2	Generate motivation to promote MVPA
PO3	Monitor personal MVPA levels
PO4	Set long-term and short-term MVPA goals
PO5	Make specific action plans
PO6	Develop self-monitoring skills and capabilities (eg, progress feedback)
PO7	Evaluate goals and revise action plans
PO8	Address identified barriers with coping plans
PO9	Identify prompts and cues for being active
PO10	Prevent habit reversal (relapse prevention)
PO11	Engage in and maintain sufficient MVPA in daily life
PO12	Serve as positive role models and leaders
PO13	Seek support and motivation from family, friends, and colleagues to engage in MVPA

**Table 3:** Example of change objectives for performance objective PO5.

Determinant	Change objective
Knowledge	Provide employees with tools and strategies for effective time management, and training and guidance on setting specific, measurable, achievable, relevant, and time-bound goals aligned with their desired outcomes
Self-efficacy	Enhance employees’ belief in their ability to create and execute action plans by enhancing confidence and providing support through skill-building exercises and positive reinforcement
Self-monitoring	Assist individuals in developing self-monitoring strategies to track their progress and adherence to action plans, such as using activity trackers, journals, or digital apps
Social support	Enhance peer interaction, foster a more supportive and collaborative environment, and encourage action planning with peers, colleagues, and family

### Step 3: Intervention Design

We designed a 3-group RCT consisting of a web-based intervention group, a blended intervention (web-based intervention combined with e-workshops) group, and an active control group. Web-based interventions are widely adopted for their accessibility, convenience, and ability to provide personalized content; however, these interventions are often hindered by high dropout rates and limited interactivity, which may reduce user engagement and effectiveness [33]. In contrast, workplace-based live workshops offer direct knowledge transfer, support, and interaction, which can enhance motivation and engagement [34-37]. By integrating these methods, the blended intervention aimed to combine the strengths of both approaches, improving its overall effectiveness. Additionally, insights from focus group discussions were considered when designing the intervention. Participants expressed a preference for textual content supported by a few simple and essential images, which informed the design of the web-based materials.

After specifying the change objectives, appropriate theoretical change methods were selected from the literature review [19]. To address the intention-behavior gap, our intervention incorporated dual-process theories, combining reflective (eg, deliberate self-regulation) and automatic (eg, habit formation) components [38,39]. Three complementary strategies, motivation, self-regulation, and habit formation, were integrated to enhance MVPA levels and sustained engagement. These strategies were operationalized using BCTs to ensure coherence, usability, and effectiveness [40,41]. Regarding the web-based intervention content, a detailed breakdown of the 6 sessions, including the timelines, questions asked, personalized feedback, and BCTs used, was provided (Multimedia Appendix 5). For example, in session 3 (week 5), action planning (1.4) was implemented by encouraging participants to articulate their MVPA goals by answering specific prompts, such as “What activity will you do?”, “Where will you do it?”, and “When will you do it?” Participants were further instructed to document their plans and place them in a visible location as regular reminders. Additionally, feedback on the evaluation and adjustment of action plans (eg, reviewing goal progress and refining prompts) helped reinforce participants’ motivation and habit development.

The control group was designed as an active control, incorporating 3 BCTs: information about health consequences (5.1), social and environmental consequences (5.3), and emotional consequences (5.6) [42]. A total of 18 essays containing general, nonpersonalized information were provided to participants. Three essays were delivered every 2 weeks across 6 sessions. The essays covered a variety of topics, such as the recommended MVPA levels, the health and productivity benefits of MVPA, the importance of time management for staying active, strategies for injury prevention, and the advantages of leading an active and healthy lifestyle. The content was factual and designed to be informative, focusing on general knowledge rather than individualized guidance.

The topics in the e-workshops for the blended intervention included understanding MVPA, building self-efficacy, establishing habits, setting goals, developing action plans, identifying barriers, creating coping strategies, using prompts, and fostering social support. Each topic was carefully designed to address participants’ needs and challenges, providing a comprehensive framework to encourage long-term engagement in MVPA. To enhance the effectiveness of these workshops, 4 additional BCTs were applied: problem-solving (1.2), instruction on how to perform the behavior (4.1), reattribution (4.3), and mental rehearsal of successful performance (15.2) [42]. For instance, problem-solving was integrated into sessions focused on identifying barriers and creating coping strategies, helping participants develop “if-then” plans (eg, “If I have only 15 minutes during lunch, then I will take a brisk walk around the office building”). Similarly, mental rehearsal was used to support participants in visualizing success when setting goals or enacting action plans, boosting their confidence and motivation. By embedding these BCTs within the core topics, the intervention ensured that theoretical strategies were effectively applied to practical, real-life situations. This design not only reinforced behavioral changes but also provided interactive and personalized elements to address participants’ unique challenges.

A 12-week intervention period was selected, as it aligns with durations commonly used in prior web-based physical activity studies. This timeframe is generally considered sufficient for participants to adapt to new exercise routines and form habits, which research suggests may take a median of 66 days [43,44]. A 12-week follow-up period was included to assess the maintenance of behavioral changes and monitor any health outcomes resulting from the intervention. These collaborative design efforts enhanced the practicality of the intervention and participants’ motivation to sustain their plans.

### Step 4: Intervention Production

**Figure 1:**
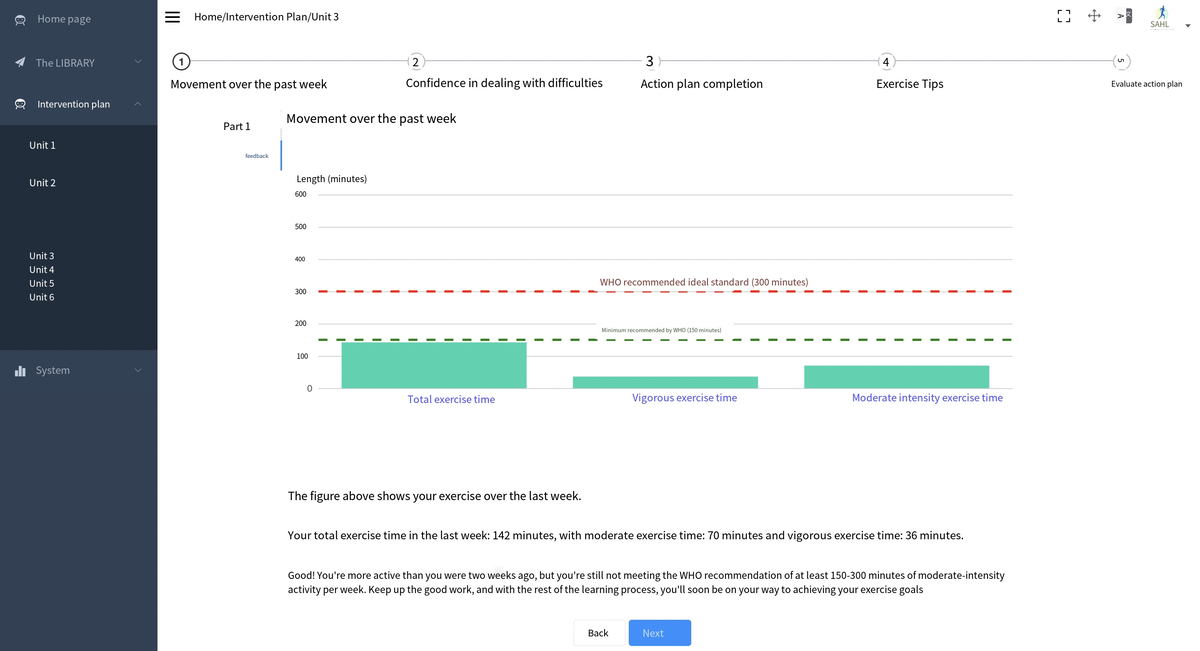
A screenshot of session 3 in the “Intervention” subsection on the website (translated from Traditional Chinese by “YouDao” translator).

We developed materials and messages for the web-based intervention that were informed by the outcomes of the first 3 steps and organized them into 6 distinct sessions. These sessions were deployed every 2 weeks via the website named “Smartly Active for Health and Life.” For reference, a screenshot of session 3 from the “Intervention” subsection has been translated from Traditional Chinese using YouDao Translate (NetEase Youdao; Figure 1). The website was structured into 2 main subsections: an “Intervention” subsection and a “Library” subsection. The participants in the blended intervention group and the web-based intervention group were able to access both subsections, whereas the participants in the control group were only able to access the “Library” subsection. Eighteen essays were included in the “Library” subsection, with 3 essays available in each session. The e-workshops were delivered in weeks 2, 4, and 8.

The planning group scrutinized and refined all materials before launching the pilot study. Recruitment posters were disseminated on social media platforms, including WeChat (Tencent Holdings Ltd), Instagram (Meta Platforms, Inc), and Facebook (Meta Platforms, Inc). A total of 18 participants were recruited for the pilot, of whom 15 (83.3%) completed the study and provided feedback. The retention and engagement rates were 83.3% (15/18) and 73% (92/126), respectively, demonstrating the intervention’s feasibility and acceptability. Participants rated the intervention highly in terms of usefulness (13/15, 86.7%), enjoyability (13/15, 86.7%), and time acceptability (14/15, 93.3%).

Participant feedback led to minor improvements, such as adding navigation aids to the website and redesigning the “Library” section by replacing long text blocks with clickable links to enhance usability and engagement. Observations during trial implementation also captured implementers’ perceptions, focusing on trialability, observability, and potential challenges under COVID-19 pandemic conditions. On the basis of these findings, strategies such as implementer training plans were developed to improve the intervention’s acceptability, implementation, and sustainability.

## Discussion

### Principal Findings

In this study, we developed a blended intervention to increase MVPA among physically inactive office employees using IM steps 1 to 4. The main outputs were (1) a logic model of the problem and a set of key determinants identified through the needs assessment, (2) POs and change objectives mapped to these determinants, (3) a set of theory- and evidence-informed intervention components and materials delivered in a blended format, and (4) feedback during the optimization and pilot phase used to refine the content and delivery of the intervention.

Our selection of IM as our guiding framework significantly influenced our findings and led us to gain critical insights into the final outcomes and the development of our interventions [16]. In step 1, we conducted a needs assessment primarily using a literature review and focus group discussions. We also formed a planning group comprising professionals from various disciplines and key stakeholders. This led to the generation of a comprehensive, acceptable, and resource-effective intervention plan. It also promoted consensus among the members, leading to a unified approach to the implementation of the intervention. Moreover, the needs assessment revealed knowledge gaps about MVPA and attitudes that could potentially hinder engagement in MVPA, which shaped the educational and motivational components of our intervention. The information gathered was vital in tailoring the intervention to meet the unique needs of our target population, thereby enhancing its relevance and value. In step 2, we ensured that the change objectives aligned closely with the identified determinants and intervention goals. This alignment was crucial to the success of the intervention as it ensured that the approach was focused on achieving meaningful outcomes while meeting specific needs. In step 3, we translated the broad goals and objectives identified in the previous steps into concrete strategies for change. These strategies formed the backbone of our intervention, providing a clear road map for its implementation. In step 4, intervention components and materials were produced and subjected to several rounds of feedback and revision. This iterative process ensured the quality and appropriateness of our intervention. Additionally, the pilot study results indicated high levels of acceptance and satisfaction among the participants, suggesting that our intervention was well received and had achieved their intended goals. Our study aligns with a growing body of research that has used the IM framework for developing disease prevention interventions [15]. For instance, a previous study used IM to create a workplace health promotion intervention targeting enhanced presenteeism. It was found that the systematic nature of IM enabled the unique needs and preferences of workers to be addressed, which resonates with our own results [45]. Nevertheless, although IM is a systematic process for intervention development, outcomes can vary widely depending on numerous factors. This reinforces the need for continued research to refine and optimize health interventions in diverse settings.

The strengths of this study lie in its systematic and theory-driven approach. Using the IM framework, we integrated stakeholder insights and behavioral theories to design an intervention supporting both the initiation and maintenance of MVPA. The flexibility of the framework during the COVID-19 pandemic allowed adaptations to online sessions and home-based exercise, ensuring feasibility and relevance in dynamic conditions.

However, despite these strengths, several limitations should be noted. First, the IM framework, while robust and comprehensive, is highly resource-intensive, requiring significant time, expertise, and financial resources, making it challenging to replicate in resource-limited settings [12,46,47]. Second, the needs assessment relied on a small focus group sample (n=12), limiting the representativeness of findings and potentially overlooking diverse perspectives [45]. Additionally, since participants were individuals who did not meet WHO’s MVPA guidelines, the findings may not generalize to populations already achieving recommended activity levels. Third, COVID-19 pandemic–related adaptations, while ensuring feasibility, altered the original intervention design, limiting its generalizability to nonpandemic contexts. For instance, workplace-level components could not be implemented due to restrictions.

Our findings have important implications for advancing health behavior change interventions in both research and practice. From a research perspective, applying the IM framework strengthened the rigor and transparency of our study while facilitating replication and exploration of mechanisms that contribute to intervention efficacy. From a practical perspective, the blended intervention outlined in this study offers a feasible road map for office employees to incorporate more MVPA into their routines, thereby improving health, productivity, and organizational outcomes. Additionally, these insights could guide policymakers in creating supportive environments that encourage MVPA in workplace settings.

Future research should address the broader applicability of IM-designed interventions across diverse populations and health behaviors. Specifically, studies should explore whether such interventions are more effective than other approaches in areas like disease management, mental health, and among vulnerable groups, such as children and older adults. Incorporating multilevel strategies that address organizational and environmental components, alongside individual-level approaches, could improve ecological validity. For instance, school-based interventions could benefit from engaging stakeholders, such as parents and teachers, to scale and adapt programs effectively. Similarly, implementing cluster RCTs in real-world settings is crucial to assessing the relevance, scalability, and sustainable impact of such interventions.

### Conclusions

This study demonstrates the value of the IM framework as a systematic, stepwise tool for designing blended interventions to promote MVPA among office employees. The framework’s ability to integrate evidence and stakeholder insights while adapting to dynamic contexts, such as the COVID-19 pandemic, highlights its versatility and practical relevance. These findings underscore the framework’s potential for developing scalable, long-term health promotion programs to improve both individual well-being and workplace productivity.

Future research should expand on this work by testing IM-based interventions in diverse populations and real-world contexts to assess scalability and broader applicability. By maintaining a continuously updated evidence base, the IM framework could enhance the design and impact of interventions across changing circumstances.

## Appendix

Multimedia Appendix 1 Intervention mapping steps and tasks.

Multimedia Appendix 2 Search strategy for the literature review.

Multimedia Appendix 3 Protocol adjustments due to the COVID-19 pandemic.

Multimedia Appendix 4 A matrix of change objectives for each performance objective cross-referenced with determinants.

Multimedia Appendix 5 Timeline, questions, personalized feedback, and behavior change techniques involved in 6 web-based sessions in 2 intervention groups.

## Abbreviations

BCT: behavior change technique
IM: intervention mapping
MVPA: moderate to vigorous physical activity
PO: performance objective
RCT: randomized controlled trial
WHO: World Health Organization

## Data Availability

Data sharing is not applicable to this paper as no datasets were generated or analyzed during this study.
