# Supplementary figures and images for: Development of a Blended Physical Activity Intervention for Office Employees Using Intervention Mapping: Intervention Development Study
Source: JMIR Hum Factors. 2026 Jul 14;13:e87328. doi: 10.2196/87328 (PMC13416307; doi:10.2196/87328)

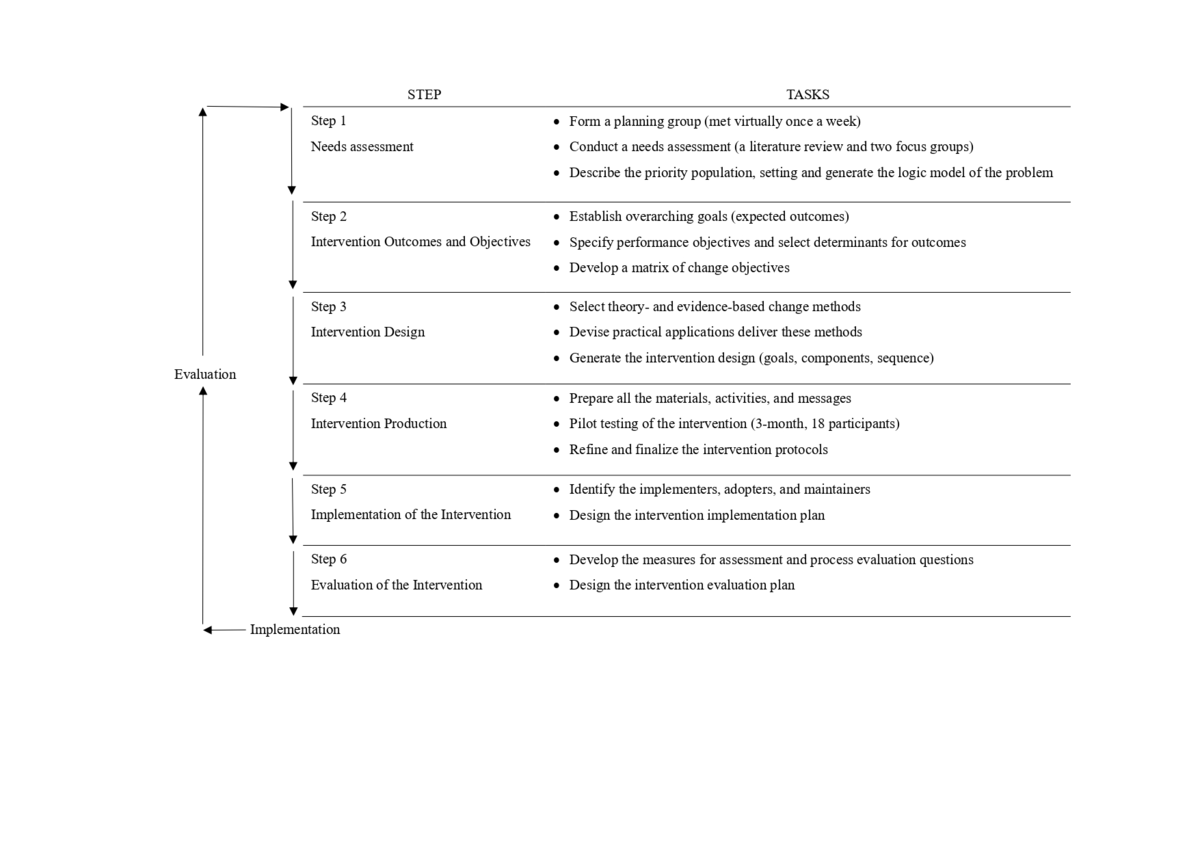

Supplement: Multimedia Appendix 1 [file humanfactors_v13i1e87328_app1.png]
