# Supplementary material for: Development of a Blended Physical Activity Intervention for Office Employees Using Intervention Mapping: Intervention Development Study
Source: JMIR Hum Factors. 2026 Jul 14;13:e87328. doi: 10.2196/87328 (PMC13416307; doi:10.2196/87328)
Supplement: Multimedia Appendix 2 [file humanfactors_v13i1e87328_app2.docx]

**Appendix 2**

**Databases**

Searches were conducted in the following databases: Scopus, PsycINFO, PubMed, EMBASE (Excerpta Medica Database), Web of Science, and SPORTDiscus.

**Search Criteria**

Time frame: Articles published before 30 July 2022.

Language: English only.

Article type: Peer-reviewed articles. Non-peer-reviewed literature (e.g., conference abstracts, book chapters, grey literature) was excluded.

**Search Terms and Boolean Logic**

Search terms were grouped into the following main conceptual categories. Boolean operators (AND/OR) were used to combine the terms, adjusted for database-specific syntax. Keywords were searched in the titles, abstracts, or subject headings.

**Physical Activity and Physical Inactivity**

physical activity
physically active
exercise
aerobic training
exercise training
resistance training
leisure-time physical activity
workplace physical activity
sports participation
sedentary lifestyle
physical inactivity
physically inactive
insufficient physical activity
insufficiently active
prolonged sitting

**Prevalence**

prevalence

**Population**

employee
worker
workplace
workforce
office worker
working-age population
desk-based employee
sedentary worker
professional staff
organizational staff
corporate health
occupational health

**Determinants**

determinant
facilitator
barrier
motivator
challenge
risk factor
preventive factor
behavior driver
influencing factor
enabler

**Health-Related Outcomes**

health
chronic disease
disease
BMI
body mass index
blood pressure
hypertension
cardiovascular disease
lipid profile
cholesterol
triglycerides
blood sugar
diabetes
metabolic syndrome
cardiometabolic health
obesity
fatigue
mental health
depression
anxiety
stress
well-being
quality of life

**Work Productivity and Related Outcomes**

work productivity
job performance
workplace engagement
presenteeism
absenteeism
workplace satisfaction

**Interventions**

intervention
health intervention
health promotion
lifestyle intervention
behavioral intervention
digital intervention

internet-based intervention
web-based intervention
app-based intervention

blended intervention
tailored intervention
workplace intervention
health education
workplace prevention

**Theory-Based Interventions**

theory-based intervention
theoretical framework
behavior change theory
self-determination theory
social cognitive theory
health belief model
transtheoretical model
social ecological model
intervention mapping
dual-process theory
goal-setting theory
habit formation model
volitional control
motivational theories
