# Supplementary material for: Development of a Blended Physical Activity Intervention for Office Employees Using Intervention Mapping: Intervention Development Study
Source: JMIR Hum Factors. 2026 Jul 14;13:e87328. doi: 10.2196/87328 (PMC13416307; doi:10.2196/87328)
Supplement: Multimedia Appendix 3 [file humanfactors_v13i1e87328_app3.docx]

**Appendix 3**

Table 1 Revised research protocol items in response to the COVID-19 pandemic

| Item | Research protocol | Revised protocol |
| --- | --- | --- |
| Study design | Cluster-RCT | RCT |
| Follow-up duration | 9 months | 12 weeks |
| Recruitment of participants | Randomly recruit eligible companies first; then invite eligible participants | Convenience sampling |
| Sample size | 495 participants (considering the cluster in sample size estimation) | 117 participants (without consideration of the cluster) |
| Planning group members | Including managers | Excluding managers |
| Needs assessment | Including in-depth interviews with managers | Excluding in-depth interviews with managers |
| Intervention content | Including environmental changes at workplace (i.e., management supporting letters and posters at workplace) | Excluding environmental changes at workplace |
| Delivery mode of Intervention  (in the blended group) | Face-to-face workshop | Online workshop via ZOOM meetings |
| Outcome measures | Fasting blood glucose (assessed at workplace in morning time); | Hemoglobin A1C (HbA1c) Test (assessed at our lab at any time of a day); |
|  | Absenteeism (records collected from study companies) | Excluding absenteeism |

RCT: randomized controlled trial
