# Supplementary material for: Development of a Blended Physical Activity Intervention for Office Employees Using Intervention Mapping: Intervention Development Study
Source: JMIR Hum Factors. 2026 Jul 14;13:e87328. doi: 10.2196/87328 (PMC13416307; doi:10.2196/87328)
Supplement: Multimedia Appendix 4 [file humanfactors_v13i1e87328_app4.docx]

**Appendix 4**

Table 2. A matrix of change objectives for each performance objective cross-referenced with determinants

| Performance Objectives | Determinants | | |  |
| --- | --- | --- | --- | --- |
|  | Knowledge, awareness and skills | Motivation and self-efficacy | Self-monitoring | Social support |
| PO.1 Possess the knowledge of MVPA, and its impacts | Provide individuals with accurate and up-to-date information about MVPA, including its definition, recommended guidelines, and the health benefits associated with regular participation. | Build confidence in ability to find reasons of being sufficiently active |  |  |
| PO.2 Generate motivation and interest to promote MVPA |  | Foster individuals' internal motivation for MVPA by highlighting the personal benefits, enjoyment, and satisfaction derived from being physically active. |  |  |
| PO.3 Monitor their own MVPA levels | Increase individuals' awareness of their current MVPA levels and the importance of monitoring their activity for overall health and well-being. | Enhance individuals' belief in their ability to monitor and track their MVPA levels effectively by providing guidance, training, and ongoing support. | Enhance individuals' ability to accurately and consistently track their MVPA levels through the use of tools such as activity trackers, mobile applications, or activity logs. |  |
| PO.4 Set long-term and short-term MVPA goals | Educate employees about the knowledge and strategies about goal-setting | Enhance employees' belief in their ability to set and achieve MVPA goals |  |  |
| PO.5 Make specific action plans | Provide employees with tools and strategies for effective time management, and training and guidance on how to set specific, measurable, attainable, relevant, and time-bound (SMART) goals that align with their desired outcomes. | Enhance employees’ belief in their ability to create and execute action plans by building confidence and providing support through skill-building exercises and positive reinforcement. | Assist individuals in developing self-monitoring strategies to track their progress and adherence to the action plans, such as using activity trackers, journals, or digital apps. | Enhance peer interaction, foster a more supportive and collaborative environment, make action plans with peer, colleagues, and family |
| PO.6 Grasp self-monitoring skills and capability (e.g., progress feedback) | Provide individuals with information and education about self-monitoring techniques, tools, and the benefits of progress feedback in tracking their performance and making adjustments. | Enhance individuals' belief in their ability to engage in self-monitoring and utilize progress feedback effectively by providing support, encouragement, and opportunities for practice and reflection. | Offer training and guidance on how to effectively self-monitor their behaviours, track progress, and interpret and use feedback to inform their actions. |  |
| PO.7 Evaluate goals and revise action plans |  |  | Encourage individuals to engage in introspection and self-assessment to evaluate their progress, identify areas of success, and pinpoint areas that may require adjustment. |  |
| PO.8 Address identified barriers with coping plans | Enhance individuals' problem-solving abilities to identify barriers, analyse their underlying causes, and generate effective coping strategies and action plans. | Foster individuals' belief in their ability to address barriers and implement coping plans, strengthening their confidence in their problem-solving skills and capacity for success. |  | Facilitate the formation of support networks and encourage individuals to seek assistance from friends, family, or professionals who can provide guidance, encouragement, and practical help in overcoming barriers. |
| PO.9 Identify the prompts/cues of being active | Enhance individuals' self-awareness of their personal triggers and cues that can motivate them to engage in physical activity, such as specific thoughts, emotions, or daily routines. | Boost individuals' belief in their ability to recognize and respond to prompts or cues for physical activity, providing them with strategies and support to build confidence in their capacity to be active. |  |  |
| PO.10 Prevent habit reversal (relapse prevention) | Cultivate mindfulness practices that help individuals stay present and aware of their actions, thoughts, and emotions, enabling them to make conscious choices aligned with their desired habits. |  | Encourage individuals to track and monitor their habits regularly, fostering awareness of their progress and potential deviations from the desired habit. |  |
| PO.11 Engage and maintain sufficient MVPA in routine life | Help individuals effectively manage their time and prioritize MVPA within their daily routines, assisting them in finding opportunities to incorporate physical activity into their schedule and overcome time-related barriers. | Foster individuals' belief in their ability to engage in and maintain MVPA by providing them with the necessary knowledge, skills, and support to overcome barriers and build confidence. |  |  |
| PO.12 Serve as positive role models and leaders of being active | Provide individuals with a comprehensive understanding of the physical, mental, and social benefits of regular physical activity, equipping them with the knowledge to educate and persuade others. | Enhance individuals' belief in their ability to be physically active and maintain a healthy lifestyle, enabling them to lead by example and inspire others through their own actions. |  | Enable individuals to leverage their social networks and relationships to positively influence and encourage others to engage in physical activity, fostering a supportive and motivating environment. |
| PO.13 Seek support and motivation from family, friends, and colleagues to engage in MVPA | Develop individuals' communication skills to effectively express their MVPA goals and needs to their family, friends, and colleagues, fostering understanding and support from others. | Enhance individuals' belief in their ability to seek support and motivation, empowering them to proactively reach out to others, ask for help, and engage in collaborative efforts to engage in MVPA. |  | 1. Encourage individuals to actively seek and engage with supportive individuals in their social network who can provide encouragement, accountability, and practical assistance in their MVPA efforts. 2. Promote the establishment of positive social norms around MVPA within the individual's social circles, creating an environment where being physically active is seen as the norm and valued by others. |

MVPA: moderate-to-vigorous intensity physical activity; PO: performance objective
