# Supplementary material for: Development of a Blended Physical Activity Intervention for Office Employees Using Intervention Mapping: Intervention Development Study
Source: JMIR Hum Factors. 2026 Jul 14;13:e87328. doi: 10.2196/87328 (PMC13416307; doi:10.2196/87328)
Supplement: Multimedia Appendix 5 [file humanfactors_v13i1e87328_app5.docx]

**Appendix 5**

Table 3. Timeline, questions, personalized feedback, and behavior change techniques (BCTs) involved in 6 web-based sessions in 2 intervention groups^a^.

| Session (time) | Questions asked | Personalized feedback and theoretical strategy involved | BCTs involved and name (code)^b^ |
| --- | --- | --- | --- |
| Session 1 (week 1) | - MVPA^c^ in the past week - Goal setting - MVPA self-efficacy - Preferred MVPA routine - Habit strength of MVPA | - MVPA guideline related to the main motivation selected (motivational strategy) - Feedback on current MVPA level related to the main motivation (self-regulation strategy) - The benefits of MVPA for physical and mental health (motivational strategy) - Strategies to increase MVPA to meet the WHO^d^ recommendations based on self-efficacy level (self-regulation strategy) - Developing habits based on preferred activity in routine life (habit development strategy) - Recognizing prompts related to habit strength level (habit development strategy) | - Goal setting (behavior; 1.1) - Feedback on behavior (2.2) - Information about health consequences (5.1) - Information about emotional consequences (5.6) - Behavior substitution (8.2) - Prompts and cues (7.1) - Habit formation (8.3) - Verbal persuasion about capability (15.1) - Instruction on how to perform the behavior (4.1) |
| Session 2 (week 3) | - MVPA in the past week - Weight - MVPA goals (both long- and short-term, open questions) - Habits and prompts - Action planning phrased with or without reference to habit (open answers) | - MVPA guidance refresher based on the main goal (self-regulation strategy) - MVPA progress feedback (self-regulation strategy) - Long- and short-term specific, measurable, achievable, relevant, and time-bound goals (motivational strategy) - Feedback on developing habits and noticing prompts (habit development strategy) - Action plan (self-regulation strategy) - Action plan with reference to behavior repetition in a stable context (habit development strategy) | - Goal setting (behavior; 1.1) - Goal setting (outcome; 1.3) - Action planning (1.4) - Feedback on behavior (2.2) - Self-monitoring of behavior (2.3) - Prompts and cues (7.1) - Habit formation (8.3) - Graded tasks (8.7) - Behavioral practice and rehearsal (8.1) |
| Session 3 (week 5) | - MVPA in the past week - Weight - Coping self-efficacy - Action plan completed - Main prompts - Action plan evaluated (open answers) | - MVPA progress feedback (self-regulation strategy) - Boosting confidence and staying motivated based on identified barriers (motivational strategy) - Evaluation and adjustment of action plan (self-regulation strategy) - Noticing prompts (habit development strategy) - Action plan (self-regulation strategy) - Action plan with reference to behavior repetition in a stable context (habit development strategy) | - Action planning (1.4) - Review behavior goals (1.5) - Discrepancy between current behavior and goal (1.6) - Review outcome goals (1.7) - Monitoring of emotional consequences (5.4) - Anticipated regret (5.5) - Information about emotional consequences (5.6) - Graded tasks (8.7) - Prompts and cues (7.1) - Habit formation (8.3) |
| Session 4 (week 7) | - MVPA in the past week - Weight - Positive social support - Negative social support - · Influence of others | - MVPA progress feedback (self-regulation strategy) - Positive influence of others and dyadic plans (motivational strategy) - Positive influence of others and dyadic plans (self-regulation strategy) - Positive influence of others and dyadic routines (habit development strategy) - Negative influence of others and staying motivated when others are not supportive - Encouraging others to be active | - Review behavior goals (1.5) - Discrepancy between current behavior and goal (1.6) - Review outcome goals (1.7) - Social support (practical; 3.2) - Social support (emotional; 3.3) - Information about others’ approval (6.3) - Focus on past success (15.3) - Self-reward (10.9) - Restructuring the social environment (12.2) |
| Session 5 (week 9) | - - MVPA in the past week   - Weight   - Behavioral barriers   - Coping self-efficacy   - Experience of habit development   - Reevaluating intervention goals (open questions) - Habit development | - - MVPA progress feedback (self-regulation strategy)   - Maintaining positive habits—coping planning (habit development strategy)   - Relapse prevention based on the main barrier selected with an emphasis on staying motivated (motivational strategy)   - Relapse prevention based on the main barrier selected with an emphasis on staying motivated and self-regulating (self-regulation strategy) - Relapse prevention based on the main barrier selected with an emphasis on staying motivated and self-regulating and maintaining positive habits (habit development strategy) | - - Problem-solving (1.2)   - Reduce prompts and cues (7.3)   - Behavior substitution (8.2)   - Habit reversal (8.4)   - Habit formation (8.3)   - Reducing negative emotions (11.2)   - Avoidance or reducing exposure to cues for the behavior (12.3)   - Review outcome goals (1.7) - Behavioral practice and rehearsal (8.1) |
| Session 6 (week 11) | - - MVPA in the past week   - Weight - Habit strength | - - MVPA progress feedback with graph (self-regulation strategy)   - Weight changes throughout the intervention with a graph (self-regulation strategy) - Tips to stay motivated (motivational strategy), to self-regulate MVPA (self-regulation strategy), and to follow newly developed routines (habit development strategy) | - - Feedback on behavior (2.2)   - Feedback on outcomes of behavior (2.7)   - Information about health consequences (5.1)   - Information about emotional consequences (5.6)   - Information about others’ approval (6.3)   - Behavioral practice and rehearsal (8.1) - Habit formation (8.3) |

^a^The table is modified from Table 1 in the original published research protocol [17].

^b^Names and codes of BCTs were drawn from Michie et al [42].

^c^MVPA: moderate to vigorous physical activity.

^d^WHO: World Health Organization.
